# Emerging Profiles for Cultured Meat; Ethics through and as Design

**DOI:** 10.3390/ani3030647

**Published:** 2013-07-26

**Authors:** Cor van der Weele, Clemens Driessen

**Affiliations:** Applied Philosophy Group, Wageningen University, P.O. Box 8130, 6700 EW Wageningen, The Netherlands; E-Mail: clemens.driessen@wur.nl

**Keywords:** meat, *in vitro* meat, animal welfare, stem cells, sustainable consumption, tissue engineering, design, ethics

## Abstract

**Simple Summary:**

The idea of cultured meat is to grow meat from animal cells with tissue engineering techniques. Cultured meat is an idea under investigation that will not be ready for the market for several years. It is also still open what it could or should be like. We argue that this openness offers the opportunity to explore different directions in which this idea could be developed. Feelings, critical thinking and the imagination all have important roles to play in this exploration.

**Abstract:**

The development of cultured meat has gained urgency through the increasing problems associated with meat, but what it might become is still open in many respects. In existing debates, two main moral profiles can be distinguished. Vegetarians and vegans who embrace cultured meat emphasize how it could contribute to the diminishment of animal suffering and exploitation, while in a more mainstream profile cultured meat helps to keep meat eating sustainable and affordable. In this paper we argue that these profiles do not exhaust the options and that (gut) feelings as well as imagination are needed to explore possible future options. On the basis of workshops, we present a third moral profile, “the pig in the backyard”. Here cultured meat is imagined as an element of a hybrid community of humans and animals that would allow for both the consumption of animal protein and meaningful relations with domestic (farm) animals. Experience in the workshops and elsewhere also illustrates that thinking about cultured meat inspires new thoughts on “normal” meat. In short, the idea of cultured meat opens up new search space in various ways. We suggest that ethics can take an active part in these searches, by fostering a process that integrates (gut) feelings, imagination and rational thought and that expands the range of our moral identities.

## 1. Introduction

Eating meat is losing moral credibility in many affluent societies. In March 2012, the New York Times noted that vegetarian and vegan arguments are increasingly dominant in moral discussions on food and that carnivores have had “surprisingly little” to say in response. The journal invited readers to partake in a contest to make the strongest possible moral case for eating meat, with a “veritable murderer’s row of judges” (Peter Singer, Michael Pollan, Mark Bittman, Jonathan Safran Foer and Andrew Light—several of whom are outspoken vegetarians). The winning essay points out that we need to realize that life and death on this planet are inevitably interwoven and that we need to choose ethically raised food and give thanks for it [1]. The public voted for the essay whose author was about to eat meat for the first time in 40 years because “the very first laboratory-grown hamburger is to make its debut”, real meat “without the mess and the misery” [2].

This laboratory-grown hamburger is a specific form of a more general idea that is known as cultured meat or *in vitro* meat [3]: making meat from animal (stem) cells, with tissue engineering techniques or through 3D printing and with animals only figuring as the source of an initial biopsy. Cultured meat does not exist yet, but its moral promises are great and manifold. As the essay-writer emphasizes, cultured meat (ideally) does not involve the raising and killing of animals and thus offers great hopes for the reduction of animal suffering in this world. But there is more. Life cycle analysis (LCA), on the basis of the expectation that it will be possible to grow cultured meat on a medium made of algae, has predicted huge environmental gains [4]. Compared with conventionally produced European beef, energy use will be reduced by 45%, greenhouse gas emissions by 96%, land use by 99% and water use by 96%, according to this tentative LCA. And there is still more. As a result of the gains in land use, cultured meat could also greatly contribute to the challenge to feed a future world of nine billion people and still allow for large areas of land to be conserved as nature or to be returned to a wild state, with more space for wild plants and animals as a result. And finally, cultured meat might lead to health gains in several ways: control for bacteria and viruses may be more reliable in cell cultures than in animals, and cultured meat might be enriched with healthy components.

This list of estimated pros in principle makes for a compelling argument to research and develop this form of protein, assuming a significant proportion of consumers would be willing to eat it. Historically it has become clear, however, that the anticipation and adoption of new technologies tends to be a more complex process, especially if these technologies imply significant shifts in societal practices—in this case for instance concerning forms of land use, reorientation of rural economies, human animal relations, the profession of animal farming and traditional forms of preparing and eating. The process of development and adoption is therefore likely to be a dynamic affair with wider cultural ramifications than merely shifting consumer attitudes towards cultured meat (*cf.* [5]).

In this paper, we will consider different moral approaches to cultured meat and make a case for an integration of ethics and design as a means to deal with the dynamic character of ongoing cultural appreciation of this new potential option. A brief historical overview first illustrates how moral considerations have been guiding the emergence of cultured meat as a technological promise: Its development appears to be a matter of moral pull more than technology push. After that, and starting with the fact that it is still very open what cultured meat might be, as a product and as a practice, we will discuss and compare three emerging moral profiles for cultured meat. The first two profiles can be discerned in existing public debates, while the third emerged from a workshop we held on cultured meat, design and ethics. In presenting it, we will argue that imaginative forms of ethics are needed to fully think through possible futures, not only for cultured meat but for meat and “protein practices” more generally. We will suggest that the mere idea of cultured meat, by undermining the inevitable link between meat and animals, may stimulate more creative and less polarized societal searches for solutions to the problems of meat 

The overall aim of the paper is to connect historical, empirical, methodological and reflexive considerations as elements of a plea for imaginative forms of ethics. Those elements are not taken up in a straightforwardly structured line of argument; we rather hope to clarify from different angles and beginnings how the idea of cultured meat inspires a rethinking of meat and how it can widen the search space for new protein practices. *Ethics through and as design* is our characterization of the type of ethics that strengthens these searches—and that meanwhile also helps to expand the range of our possible moral identities.

## 2. Cultured Meat and its Moral Promise

Early in the 20th century, cultured meat was proposed by a few visionaries. Churchill was one of them; in his essay *Fifty years hence* [6], he remarked that “we shall escape the absurdity of growing a whole chicken in order to eat the breast or wing, by growing these parts separately under a suitable medium”. This prediction was part of a broad-ranging science fiction-like vision of future improvements in many spheres of life. Some decades later, Willem van Eelen was another visionary who, after having known severe hunger in Asian internment camps during the Second World War, was brooding on new forms of food production [7]. Van Eelen is still alive and hopes to live to see cultured meat replace factory farming, something for which he turns out to need patience as well as longevity; only in recent years has the idea started to gain some momentum.

In 2003, the Australian artists Oron Catts and Ionat Zurr (*cf.* [8]) succeeded in keeping muscle tissue from a frog alive for three months and let it grow somewhat. The project, called *Disembodied Cuisine*, yielded very tiny steaks that were marinated in calvados, fried in honey and garlic and served as pieces of ‘victimless meat’ during a dinner performance in a museum in Nantes, France. The donor frog was alive, and (allegedly) present at the performance. In 2004, Jason Matheny, who was at that time a student of public health, was alarmed by what he learned about meat production and founded *New Harvest*, an organization that has been promoting research on cultured meat ever since [9]. Matheny’s influence played a role in getting a four-year research project off the ground in the Netherlands, which may perhaps count as the first serious research effort into the development of cultured meat [9]. The project, led by Henk Haagsman of Utrecht University, ran from 2005 to 2009 and did produce four PhD theses, but also made it clear how far removed from a viable product this research still was. One of us is involved in an interdisciplinary successor-project [10]. Overall, money for cultured meat research has so far been hard to obtain. 

Meanwhile, attention for global problems of meat has further increased —the Food and Agricultural Organization (FAO) report *Livestock’s long shadow* [11] added environmental problems to the agenda in 2006—and has sparked further societal interest in cultured meat. In 2008, the American organization People for the Ethical Treatment of Animals (PETA) announced a one million dollar prize to whomever would be able to make *in vitro* chicken meat, indistinguishable from real chicken meat, and sell it commercially by June 30, 2012 [12]. Though PETA extended the deadline a bit, nobody claimed this prize. In 2011, Professor Mark Post of Maastricht University announced that he was aiming to offer a “proof of principle” for cultured meat with the help of existing tissue engineering technology. The attempt, which is to take the form of a hamburger, is funded by a wealthy and anonymous American, and is intended to generate attention as well as money for further research. But both Post and Haagsman estimate that even with generous funding it will take ten to twenty years before tissue engineered cultured meat is on the market. In 2011, Gabor and Andras Forgacs founded a company called *Modern Meadow* that aims to make cultured meat as well as leather with the help of 3D printing technology. 

This brief overview may suffice to illustrate that cultured meat has ceased to be merely a wild idea; it has become a source of hope in response to the increasing problems of meat, with societal voices calling for research for moral reasons. Ethicists have joined that call. Peter Singer has repeatedly been describing cultured meat as the main source of hope for the reduction of animal suffering. In 2008, Hopkins and Dacey [13] were the first to review moral arguments for and against cultured meat, which they framed as a potential option for people who want to eat meat yet do not want to contribute to animal suffering. The arguments for cultured meat, they say, are very clear and straightforward, and “in essence the hopeful outlook of a technological fix”; cultured meat could eliminate much animal suffering and environmental damage and it could produce healthier food. Yet there are also objections and hesitations, to which they devote the heart of the paper. Thus, they discuss worries about unnaturalness and about responses of disgust (“yuck”) as well as the idea that technological solutions amount to “moral cowardice—choosing a quick fix over genuine moral work”. In their analysis, none of the objections carries enough weight to counter the potential gains. The alleged “unnaturalness” of cultured meat may be precisely what we are looking for, since at least some of the “natural” ways of producing meat are very problematic. Disgust responses are interesting and important but can hardly count as final judgments, while the moral cowardice argument may be convincing if the purpose is to cleanse our souls, but it does not demonstrate that technology cannot be a powerful moral tool, in this case for the reduction of animal suffering ([13], p. 591). 

After weighing pros and cons they conclude that cultured meat is not just interesting, but “something we may be morally required to support”. They also note, more generally, that morality does not necessarily have a responding role to new technology; it can also be guiding: It may “champion and assist” in the development of technologies as steps towards a better world. 

## 3. What Is Cultured Meat Going to Be: Two Profiles

Hopkins and Dacey’s weighing of pros and cons is so clear that it may look like there is not much more for ethics to consider for the time being, except if one disagrees with them. Yet their argument at this point only functions as an argument for research on cultured meat, not (yet) for cultured meat as a product, a production process or a food practice. None of these exist, and all of these may take very different forms. As sociologist Neil Stephens has put it, cultured meat is an “as yet undefined ontological object” [14]. Building on this insight, we think the future of cultured meat can still largely be seen as an ontological void. We agree with Hopkins and Dacey that the role of ethics to “champion and assist” in the development of a new technology is an important and sometimes underrated one. In our view, that role requires moral imagination, for venturing into the void, filling it in and thinking through how attractive different paths are from existing and emerging moral points of view and how they might or might not align with present and future food systems and habits. This role fits in with Dewey’s pragmatist view of ethics as a form of inquiry, that is to say as a search for ways to improve the world in an open ended process which involves experimentation and an active relation to experience. Disciplinary distinctions are not fundamental and the imagination takes a central position. According to Dewey, existing moralities are bound to the *status quo*, while “the first intimations of wide and large redirections of desire and purpose are of necessity imaginative” [15,16,17]. Thinking through possible futures, in a process Dewey called “dramatic rehearsal”, includes playing with new ideas and their combination, as well as with doubts and objections, positive and negative feelings, argument and reflection. As we will see, the idea of cultured meat functions as a catalyst for wider imaginative thought on meat and its potential alternatives.

**Figure 1:**
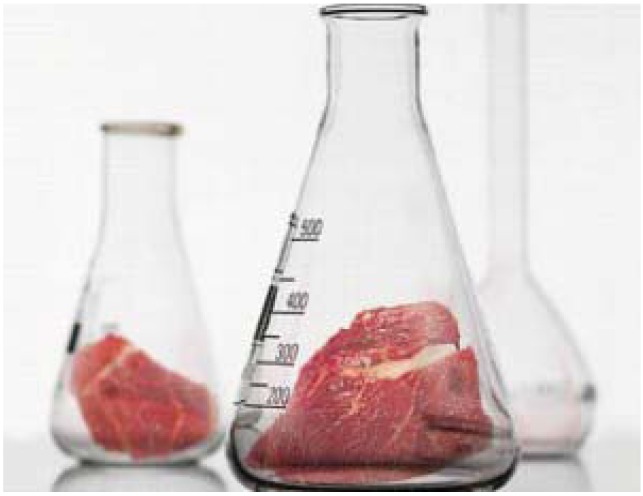
First associations: a steak in an Erlenmeyer flask.

Before we discuss our efforts to encourage imaginative thinking, let us see how the idea of cultured meat is already taking shape in research and societal debate. Thinking about cultured meat started by transplanting existing views of meat to cultured meat. Illustrations often show an ordinary piece of meat and add an “*in vitro*” element, such as in Figure 1 in which a steak is shown in an Erlenmeyer flask. 

Yet at the same time, this “*in vitro*” component turns cultured meat into something completely different in many and confusing ways: Something that is potentially good for animals, something unnatural, something scary, something to counter climate change and pollution… all these qualifications have been elaborated on to characterize cultured meat. Within the large set of public arguments, two clear moral profiles have emerged so far, a vegetarian profile and a sustainable profile, both containing a different ambivalent mix of attraction and repulsion.

A. A “vegetarian” profile. For many people, the first and most significant characteristic of cultured meat is its promise for animals in a world that eats ever more meat. This is precisely why animal organizations tend to embrace the idea, often after some reluctance. Given the persistence of meat eating, here is a potential way to continue doing so without (significant) harm for animals. In 2008, PETA made a highly visible point of encouraging cultured meat through its announcement of a contest to make *in vitro* chicken meat. Meanwhile, the announcement said, anyone who does not know a test tube from a champagne flute can still help animals by eating the already existing vegan meats, that is, plant-based protein products. For PETA, cultured meat is a road to a vegetarian or a vegan world. The author of the New York Times essay celebrating the advent of cultured meat that won the public’s prize, turned out to be Ingrid Newkirk, director of PETA.

This approach to cultured meat has led to the criticism that it will make animals disappear from our lives. According to Simon Fairlie [18], who sees cultured meat as part of a vegan set of goals, the convergence of technological developments such as genetic manipulation, synthetic biology and cultured meat amounts to a horror scenario that will perhaps satisfy vegans but that will decisively estrange us from animals and nature. Fairlie more generally denies that renouncing meat is the best answer to the unsustainability of meat eating. Instead, he proposes to return to more traditional rural forms of agriculture, which would include small scale animal husbandry and a significant decrease in meat consumption but explicitly excludes new technologies such as cultured meat.

B. A sustainable profile. Global meat consumption is very unsustainable. This can quantitatively be illustrated in many ways, starting from the inefficiency of producing animal proteins from plant proteins (with conversion losses of 60% to 90%). Now that 70% of all arable land is already used for livestock production, and meat consumption is predicted to double in 40 years, anyone can do the math, says Mark Post: “you can easily calculate that we need alternatives.” That is, if we want meat to remain available and affordable: “If you don’t do anything, meat will become a luxury food and be very, very expensive.” [19].

In this profile, safeguarding future meat consumption is the goal, its (environmental and eventually economic) unsustainability is the problem, and cultured meat is the solution. In order to fulfill its sustainability promise, the aim of cultured meat development is not to satisfy vegetarians or vegans (after all they are not part of the problems associated with meat eating), but meat eaters. 

The attraction of this profile is that it aligns with mainstream and quantifiable trends of higher and higher meat consumption in a world of growing numbers of people with an increasing demand for meat. But this profile, too, has its unattractive sides, especially for those who think that the problems of meat can only really be solved by new values and new consumptive behavior. Cultured meat would allow the continuation of meat consumption, or morally problematic consumptive attitudes in general. 

Thus, different as these profiles are, in societal debate they are subject to the same criticism: We should not trust in a technological fix because it will not solve our *real* problems; let us rather aim for changes in agricultural practices, our values and/or our behavior. Hopkins and Dacey [13] responded to the related objection that looking for technological solutions amounts to moral cowardice (“choosing a quick fix over genuine moral work”) by pointing out that virtue related ideals are not the only moral goals; there is also the consequentialist goal of the reduction of animal suffering. They also remarked that technology and morality do not represent separate roads to change. It may take “a few years of living with something like cultured meat to help change our mores so that people in the future find eating meat from living animals unbearably barbaric.” We agree that technology and morality interact, and we think that for such interaction to begin in the case of cultured meat, we do not even need to wait for the existence of commercially available cultured meat; the mere idea is enough to stimulate thought on our present and future meat consumption, as we will illustrate below.

## 4. Cultured Meat Workshops: Background Ideas

Instead of judging the pros and cons of the two existing profiles, their tensions, or what they might mean for marketing, we prefer to enlarge the list of possible profiles for cultured meat. The future of food is uncertain in very many ways and the roads ahead, including the possible roads for cultured meat, deserve elaborate exploration. Tissue engineering, 3D printing and additional techniques will enable the production of other things than hamburgers, sausages or steaks; it does not require much imagination to see that cultured meat is a potentially revolutionary technology that could dramatically change established routines of production and consumption. The options afford the possibility to play, first imaginary, potentially also real, with form, color, additions and taste, as well as with various production processes, moral profiles, marketing profiles and consumer practices. It seems to us a loss of opportunity to restrict the contribution of ethics to an evaluation of arguments for and against (underdeveloped forms of) cultured meat. 

We have been exploring how ethics can help enlarge the search space for cultured meat. To that end, we set up workshops to design (and “dramatically rehearse”) moral profiles, in which we incorporated three background ideas: (a) We agree with Hopkins and Dacey that it is important to take objections to cultured meat seriously. (b) Images help in making ideas and scenarios more concrete. (c) New technologies are not just additions to life, they also change existing reality. Let us briefly explain these background thoughts and their meaning for the workshops.

a. Taking objections seriously: We tried to do that not by weighing arguments for and against, but by using objections, hesitations and aversive gut feelings as inputs for the workshop. Earlier interactions, during conversations and interactive lectures in the Netherlands [20], had made one of us (CvdW) distinguish three crude categories of first responses to cultured meat: (1) Wow, good idea! (2) Yuck, disgusting! (3) Interesting, but very technological. These categories appeared to cover first responses pretty exhaustively, in the (again crude) sense that very few people opted for the category “other responses” (Table 1).

**Table 1:** First responses to the idea of cultured meat.

First Response	Percentage (%)
Wow!	40–80
Yuck!	5–25
Interesting, but…	10–35
Other	5–10

Explanations following yuck responses at other occasions made it clear that such disgusted feelings typically came with aversive associations with “messing around with food”, in the form of genetic modification as well as industrial meat production [21]. But first reactions were often quickly followed by second ones. As one man rather characteristically remarked in the sentence following his yuck response: “But wait a minute. When I think of what it might mean for animals, it already looks different.” 

**Figure 2:**
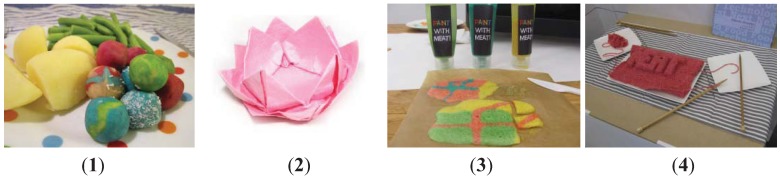
Eindhoven student designs of cultured meat.

First responses, both positive and negative, are not static. Further thoughts and feelings about cultured meat are often mixed and ambivalent; they include hope, uneasiness, distrust, curiosity and wonder. On the positive side, hopes for animals are dominant, as well as the prospect of eating meat without a troubled conscience. On the negative side, people wonder for example whether cultured meat would be meat “without a soul”, whether it could be unhealthy in slow and unexpected ways, perhaps even entail particular diseases, and whether if successful it would lead to a world without farmers and farm animals. That it is “unnatural” or “too technological” also makes people uneasy, and it sometimes provokes thoughts of large, gloomy and empty industrial terrains, or about multinational companies amassing even more power over our food (*cf.* McHugh [22]). We think such feelings and images, the negative as well as the positive ones, are meaningful starting points for further thought about cultured meat.

b. Visual design. At the University of Eindhoven, cultured meat is one of the themes in the industrial design program *Next Nature*, led by Koert van Mensvoort [23]. From the fall of 2011 onward, students of industrial design have been working on many different ideas of what cultured meat could be, what it might look like, how it might be produced, how it might be packaged and marketed, etcetera. One of us (CvdW) has been involved as a “client”, with the aim of using the finished designs as starting points to enlarge the space for thinking about cultured meat in workshops or elsewhere. Figure 2 shows some of the results. Mark Kanters’ *Magic Meat* (1) is based on the marketing idea to seduce children through magically colored balls. Frank van Valkenhoef’s *Origami meat* (2) starts from the idea that 3D printing could produce flat layers of muscle cells that might be folded in different ways. Ilse Maessen’s *Paint with meat* (3) pictures cultured meat of different colors in tubes, for the production of “paintings” to be baked in the oven, while Alberto Gruarin’s *Knit the new meat* (4) starts from the idea of a narrow thread of muscle cells with which more complicated structures might be put together.

c. Cultured meat and the meaning of meat. New technology and new morality (attitudes, behavior) are often presented as opposing roads to societal change. One of us (CD) has studied and elaborated the alternative idea that new technology often functions as an intervention in existing moral debates and in effect changes the sites and the terms of debates as well as the actors participating in them. Cultured meat, or rather the mere idea of cultured meat, is a clear case. For example, the difficulty to find a good name for cultured meat also raises questions about how to call “normal” meat in comparison. Will cultured meat make us talk about “old” and “new” meat? Willem van Eelen has been forcefully insistent that cultured meat is not going to be some peculiar kind of meat; it will simply be “meat”. Tissue engineer Mark Post also wants to ‘just call it meat’ [18]. But if so, how to distinguish it from other kinds of meat? May cultured meat perhaps lead to an ever-greater diversification, e.g., to a distinction between CM (cultured meat), FFM (factory farmed meat) and FRM (Free range meat)?

## 5. A Third Profile

Our workshops were set up on the basis of these ideas and inputs. At two conferences (one about bioethics, one about ethics and emerging technology) we used the duration of a session (90 minutes) for a workshop. After introducing the idea of cultured meat and its currently foreseen forms, we presented the visual student designs to encourage an imaginative atmosphere. They were followed by the core of the workshop: interactively thinking through (“designing”) different futures for cultured meat. It is not the aim of the present paper to present an overview of questions, considerations and directions explored during the workshops [24]. Instead we will focus on one idea, which came up during the second workshop: That in the future we might all have a pig in our backyard or in our local community, from which some stem cells are taken every few weeks in order to grow our own meat, either in a machine on our kitchen sink or in a local factory. It is an idea that in some form or another often turns up in conversations on cultured meat [25]. It typically takes the form of pigs or cows in urban farms or backyards, held as pets and serving as donors of muscle stem cells. So it was hardly surprising to see it also turn up during the workshop. What the workshop added was an opportunity to exchange thoughts and feelings and further elaborations. The degree of shared enthusiasm in response to this idea was remarkable; it was so large that the preferred future of cultured meat was completely clear, as far as the participants of this workshop were concerned. A combination of joy, inspiration and amazement characterized the atmosphere. 

What makes this idea so attractive and inspiring to at least some people? We surmise it has something to do with the unexpected combination of many good things that have always seemed incompatible. Here, all of a sudden, we get a glimpse of a possible world in which we can have it all: meat, the end of animal suffering, the company of animals and simple technology close to our homes. The pig in the backyard or in the community, that is a pet and a cell donor for cultured meat at the same time, creates the possibility of sharing the world with animals in sustainable as well as conscientious ways, while we do not have to give up eating meat. The prospect eliminates the suffering caused by intensive farming, but not by replacing it with an abolitionist world in which urban vegans are completely separated from nature and from animals, pictured by Fairlie in such bleak terms. On the contrary, in this possible world relations between humans and animals improve spectacularly. In an idealized version, it would even improve upon practices such as chickens kept in a backyard for eggs or honeybees kept on an urban rooftop, as these modes of food production require more significant interfering with the behavior and circumstances of the animals. The feeling that this prospect is almost too good to be true may help to explain the special atmosphere.

A further reflection on this shared enthusiasm is that it also fits in with several recent art and design projects that have been calling attention to the animals, notably pigs, which are the source of meat. The intention of those projects was not only or primarily to highlight welfare conditions on intensive farms and make people reflect on their choice to have animals killed for food, but also to rethink relations between humans and farm animals. For instance, ‘Peergroup’ arranged for a form of micro scale urban farming called ‘Varkenshuis’, which involved two pigs living in an urban community for half a year [26]. After having been fed on food scraps and played with in a tiny petting zoo, they were eventually slaughtered for meat that was distributed among those who had lived near them and were willing to have some. Another project that tries to re-establish relations between humans and pigs, but through modern media, is ‘playingwithpigs’, an effort to create interspecies video games for intensively farmed pigs, initiated by one of us (CD). The project intends to relieve the animals’ boredom by offering them the opportunity to play with humans [27]. These projects have been trying to draw attention to the possibility of having a meaningful relation with the animals that some of us eat. Instead of implicitly denying and ignoring the lives that are cut off to provide meat, these projects promote active reflection on deep ambivalences regarding meat and animals. Cultured meat could provide a way to ‘solve’ much of the ambivalence, which seems to have become more and more central in public experience of the past few years. 

“The pig in the backyard” can be seen as a third profile for cultured meat. While in the first two profiles cultured meat was an answer to a clear problem, animal suffering and unsustainability respectively, the third profile is different in that the pig in the yard does not seem to be an answer to one main problem: It addresses a cluster of problems, desires, solutions and objections. It may come up, for example, when you are troubled by intensive farming, but also sense there is something amiss with the abolitionist tendencies in vegan proposals. It may also arise from the desire for local and low-key technologies, which at first thought most people tend not to associate with cultured meat. In the light of enthusiasm for this profile, the thought that cultured meat is “interesting, but very technological” can now at least partly be interpreted as a worry about a further alienation from our food. The profile addresses this worry; it involves renewed and close contact with a source of our food rather than a fully industrial ecology of bioreactors and artificial flavorings. Finally, in its focus on daily life this profile foregrounds what in the other profiles is simply assumed: The allegedly ‘long and happily ever after’ living donor animal, as well as the nitty-gritty work of extracting and growing tissue. Even though many associate ‘the lab’ with crisp hygiene, a suspicion may arise that the actual process is much more messy than often portrayed. 

In short, the profile might be said to represent a strategy of “bricolage” or “tinkering” with practices, technologies, desires and motives and to rely on a diversity of sources to find new ways to solve as many problems and satisfy as many values and desires as possible. 

The three moral profiles for cultured meat we described differ in what they see as the trouble with meat, in their main goals, the way in which cultured meat is thought to be helpful and in their styles of reasoning. The following scheme wraps up these differences (Table 2).

**Table 2:** Three moral profiles for cultured meat.

	Vegetarian	Sustainable	Pig in the yard
**Meat is… **	Bad for animals, therefore bad	Desirable but unsustainable	Riddled with ambivalence
**Goal**	A vegetarian (or perhaps vegan) world	A sustainable world	A better/less alienating world
**Human animal relation**	No animal suffering or even no exploitation at all	Not relevant *per se*	Ubiquity of individual animals with personal relations to humans
**How cultured meat helps**	No animals, so no animal suffering	Large gains in sustainability	New directions and combinations
**Mode of reasoning**	Moral principles	Quantified data	Tinkering/Relational

## 6. Ethics through and as Design

It is not our intention to suggest that three profiles exhaust the possibilities. For example, human health might be conceived as the core value of a fourth moral profile, while global food security might define a fifth, and “room-for-nature” a sixth, though these might also be taken up in a sustainability perspective. But three profiles suffice to show that the “ontological void” of cultured meat can be filled in very different ways. While in the abstract it might seem that all the potential advantages of cultured meat can be combined, in practice roads diverge in different directions. As we see it, for the time being such divergence is constructive and helpful, since a more thorough exploration of possible protein futures is highly welcome. We want to end with some reflections on new alliances in this process, and on possible roles for ethics. 

The idea of cultured meat has been generating so much hope and activity that we can truly speak of a moral “pull”, even though at the same time there is also much ambivalence and probably latent resistance [28]. Since the moral attractions are so diverse, they create new options for common ground; the mere idea of cultured meat has been unsettling old positions and undermining old polarizations [29]. For example, although the main values as well as the ultimate goals of the first two profiles are different, cultured meat fits in with both perspectives. By embracing cultured meat, PETA has become more pragmatic in its search for promising leads. In the traditionally typical road to vegetarianism and veganism, individual moral awakening is followed by a process of transformation [31] which finally leads to avoidance of meat or even all animal products. Allowing for the option of cultured meat implies making room for other motivations than caring for animals, in particular love of meat [32]. It implies an acknowledgement that vegetarian ideals go against many people’s deeply entrenched habits and desires and it involves making dirty hands on the road to societal change. Those who aim for sustainable forms of meat production, on the other hand, start from a position in which they attempt to keep the search for cultured meat as mainstream as possible. Their efforts are directed at current meat eaters rather than vegetarians or vegans and they tend not to emphasize contested moral values, in particular concerning animals. Given their wish to associate cultured meat only with mainstream values, PETA’s alliance with cultured meat is an uneasy one from their perspective too. Yet, they agree that cultured meat is better for animals and the development of animal free serum has been one of the central aims of *in vitro* meat research over the past years. 

In presentations and discussions of cultured meat, extensive lists of meat problems usually figure, with animal suffering either at the top or at the bottom. Either way, such lists convey the message that meat production and consumption is highly problematic in many ways, and cultured meat has made such lists more conspicuously present in many debates beyond those oriented towards meat issues *per se*.

Cultured meat thus opens up new search space even before it exists, by stimulating both design activities and new alliances, however uneasy. Let us end by summarizing under two headings how ethics, as a form of pragmatic inquiry, may join and strengthen this search process. 

### 6.1. Sorting Out Directions and Tensions

Reflection on debates and sorting out the arguments is a traditional role for philosophy and ethics, which in this field too can be helpful to inform further debate and R&D. It may serve several goals. For example, in this paper we have been distinguishing moral profiles in existing debates, not with the aim to judge or weigh them but to compare them, add a third one and point out that the search for and development of moral profiles is far from complete. 

Debates on cultured meat always involve more than cultured meat. Most conspicuously, discussions of cultured meat incessantly interweave with arguments on (the future of) “normal” meat. Lists of meat problems that always turn up in discussions of cultured meat are an example. Earlier in the paper we mentioned another example, namely that discussions about a suitable name for cultured meat also lead to questions on how to name traditional meat [33]. Furthermore, thoughts about the alleged “unnaturalness” of cultured meat invariably lead to remarks like “come to speak of that, how natural is intensive husbandry”? [34]. The technological character of cultured meat draws attention to the technological ways in which meat and other food is currently produced and processed. And the third profile we identified illustrates this entanglement of meat and cultured meat in yet another way; the profile is not only about producing cultured meat, but at the same time addresses our relations with animals and the way we keep them in husbandry practices.

Cultured meat also inspires thoughts about other alternatives to meat and thus tends to lead to wider discussions on protein consumption and production. In these hotly debated areas, full of explicit and implicit assumptions as well as hard-boiled standpoints, cultured meat undermines existing lines of division and introduces new ones. Sorting out the turns and arguments can be helpful and important in engaging with questions on meat production and animal use. 

### 6.2. Integrating Design and Reflection

Ethical inquiry can also be part of the process of designing new options for the future in more direct ways. We held our workshops with the aim of devising moral profiles for cultured meat, on the basis of the idea that integrating the idea of new technology in society is not simply a matter of consumer “responses” or “attitudes”. They are relevant, of course; for example, widely existing ambivalences and tensions concerning meat suggest the existence of attitudinal instability. But attitudes and responses in themselves do not determine the development of new options and directions. More is needed for that: new ideas, research, concepts, combinations, creativity, design, interpretation, interaction, and reflection. Our workshops attempted to create room for these processes, by taking (gut) feelings seriously, by encouraging an imaginative atmosphere through images of the student designs, and by acknowledging and integrating how the idea of cultured meat activates as well as overthrows existing ambivalences on “normal” meat practices. In this paper we focused on an interesting outcome of the workshops (rather than on their methodological evaluation [24]): an additional moral profile for filling in the ontological void of cultured meat. In our view, this outcome exemplifies that unorthodox combinations of new technological options, moral considerations, emotional responses and societal trends are both needed and possible. The process may involve complex shifts; given the wide range of meanings of meat and its consumption, changes in options with regard to meat involve changes in our moral relations to food, animals and ourselves. Ethics as a pragmatic form of imaginative inquiry can help in these processes, for example by creating spaces of integrative activity and by reflecting on them. 

## 7. Conclusions and Outlook

Two different moral profiles can be recognized in public debates on cultured meat, one prioritizing animal welfare, the other sustainability. This paper, apart from identifying and distinguishing these profiles, has argued that the idea of cultured meat calls for a more thorough exploration of the different directions in which it might be developed. Feelings, critical thinking and imagination all have important roles to play in this process. The two workshops we organized as experimental testing grounds for integrative and imaginative moral exploration confirmed that thinking about cultured meat is also a way of seeing the ambivalences of present meat practices in a new light. The workshops yielded a third profile for cultured meat, which we called “The pig in the backyard”. 

The three profiles described here do not exhaust the options. In agreement with Dewey’s view that large redirections of desire and purpose necessarily find their start in imagination, the aim of this paper has been to illustrate how the mere idea of cultured meat acts as a catalyst for new ideas on meat and its possible alternatives, and how ethics can aim to strengthen this exploration. In the process, not only outlooks on future protein practices may change, but also outlooks on ourselves. A larger set of moral identities becomes available as cultured meat invites reflection on the many reasons one can have to consume less meat, only particular types of meat, no meat at all, or not even any animal products. Ongoing reflection on the wider implications of such choices centrally involves aesthetic and affective experiences. For example, what is an attractive landscape and what is the role of (domestic) animals in them? And what counts as meaningful relations to animals [35]?

When cultured meat one day will finally enter the market as a product, it may already have a rich history as an idea that has inspired new perspectives on old meat practices, new designs for protein practices and new views on who we are in relation to food, animals and our environments.
